# United Kingdom Veterinarians’ Perceptions of Clients’ Internet Use and the Perceived Impact on the Client–Vet Relationship

**DOI:** 10.3389/fvets.2017.00180

**Published:** 2017-10-19

**Authors:** Lori R. Kogan, James A. Oxley, Peter Hellyer, Regina Schoenfeld-Tacher

**Affiliations:** ^1^Clinical Sciences, Colorado State University, Fort Collins, CO, United States; ^2^Independent Researcher, Romford, United Kingdom; ^3^Department of Molecular Biomedical Sciences, College of Veterinary Medicine, NC State University, Raleigh, NC, United States

**Keywords:** Internet, telehealth, telemedicine, United Kingdom, veterinarian

## Abstract

The Internet is a commonly used resource for accessing health information. Despite the Internet’s popularity in the human health field, little is known about the Internet’s impact on veterinarians, their clients, and the veterinarian–client relationship. The aim of this study was to investigate the perception of veterinarians from the United Kingdom of clients’ use of the Internet and the perceived impact on pet health and the veterinarian–client relationship. A survey was distributed between January 4 and March 3, 2017, *via* an online link. In total, 100 veterinarians completed the survey. This study found that most UK veterinarians feel their clients access the Internet to find pet health information, yet often do not understand what they read online. Importantly, 40% of veterinarians stated that the Internet has a negative impact on companion animal health. This small-scale study found mixed opinions regarding veterinarians’ perceptions of their clients’ use of the Internet and the potential impact it has on the client–veterinarian relationship. Research on clients’ actual use of the Internet and their associated perceptions is a next logical step.

## Introduction

In 2016, the majority (82%) of the UK adult population used the Internet on a daily or almost daily basis, a demonstrable increase from 35% in 2006 (1). As witnessed in the human health field, the Internet is a commonly used resource for accessing health information (2). Despite the Internet’s popularity in the human health field, little is known about the Internet’s impact on veterinarians, their clients and the veterinarian–client relationship. Furthermore, the response of pet owners to information sourced online is also an area which needs to be better understood.

Previous research has highlighted the fact that pet owners frequently consult the Internet for pet health information in addition to seeking advice from their veterinarians (3, 4). However, the Internet is an extensive resource and thus there is the risk for pet owners to access inaccurate and unreliable information, potentially influencing pet health and veterinarian–client relationships (4, 5). This has been previously demonstrated, as Taggart et al. (6) evaluated 44 website related to canine cruciate ligament disease and found that the quality of website contents varied and was influenced by both terminology used and if the website was authored by a veterinarian. In addition, Hofmeister et al. (3) reviewed websites relating to anesthesia in dogs and found that these were generally found to be incomplete and even potentially misleading in relation to stated risks relating to specific breeds. Similarly, Jehn et al. (7) found that of 30 websites reviewed related to osteoarthritis in dogs and were found to be often incomplete.

One method recently explored to combat the potential of inaccurate online information is the provision of “information prescriptions” (the provision of details of an online source (such as web link or name of a website) by a veterinary professional with relevant information about pet health or other related problem) by veterinary staff. Veterinary clients in the United States have reported finding these information prescriptions informative and trustworthy (8). The aim of this study was to investigate UK veterinarians’ perception of clients’ use and understanding of pet health information on the Internet, current provision of online material by veterinarians to clients and the perceived impact of online pet health information on the veterinarian–client relationship.

## Materials and Methods

A survey was adapted from Kogan et al. (4), approved by Colorado State University Research Integrity and Compliance Review committee, and piloted. The final survey, available upon request, was distributed between January 4 and March 3, 2017, *via* an online link to a survey through social media (e.g., Facebook, Twitter, and LinkedIn), two veterinary blogs and a letter in a veterinary publication, the “Veterinary Times” [see (9)] a publication aimed at veterinary surgeons and nurses. The questionnaire covered four main areas including (i) demographics [age, sex, location, type of veterinarians (e.g., small animal)], (ii) perception of veterinarians on client Internet use and understanding of online information, (iii) frequency of veterinarians providing online information (i.e., information prescriptions) to clients and the format, and (iv) the perceived impact of the Internet on the vet–client relationship. All respondents answered all questions unless stated otherwise. As only 100 responses were received, only descriptive statistics are reported.

## Results

In total, 100 veterinarians completed the survey. The majority of respondents (65%) were female. Nearly all respondents lived and worked in England (90%). The remaining were from Scotland (6%); Wales (3%), and one respondent stated both England and Wales. A wide range of ages were reported with 57% being between 20 and 40 years, 30% between 41 and 60 years, and 13% being over 60 years of age. The majority (69%) were exclusively small animal veterinarians and 17% predominantly small animal veterinarians. Only a small proportion were equine (6%), mixed practice (5%), and exotic (3%) veterinarians.

### Veterinarian Perception of Client Online Use and Understanding

Over 70% of veterinarians thought that nearly all (>80%) clients have access to the Internet either at work or home. Fifty-seven per cent of veterinarians thought that the majority (61–100%) of their clients use the Internet to find pet health information. Yet, 73% of veterinarians thought that only a minority (0–40%) of clients understand what they read online regarding pet health information (see Table 1).

**Table 1:** Veterinarians’ responses to statements about clients’ use and understanding of the Internet (*n* = 100).

Question	0–20%	21–40%	41–60%	61–80	81–100%
What percentage of your clients do you think have access to the Internet either at home or at work for personal use?	0	0	5	24	71
What percentage of your clients do you think use the Internet to look for health info about their pets?	1	13	29	39	18
What percentage of your clients discuss the pet health information they find on the Internet with you?	17	38	30	12	3
Among your clients who access the Internet for pet health information, what Percentage do you feel understand what they read online?	38	35	20	5	2
Among your clients who access the Internet for pet health information, what percentage do you feel trust what they read online?	8	14	30	42	6

#### Information Prescriptions

The question “*How often do you suggest specific websites (not including your clinics website) to clients*” resulted in 28% stating less than once a month, 27% several times a month, 21% several times a week, 15% at least once a day, 3% many times a day, and 6% never. Of 94 veterinarians who reported they do suggest website to clients, the majority told clients the name and address of a particular website (31.9%), gave them a written copy of the website name and address (21.3%), or gave them both written and verbal instruction (17%) (see Table 2 for a full breakdown).

**Table 2:** Methods in which veterinarians recommend website to clients (*n* = 94).

Options	*n*	%
Give them a written copy of the websites name and address	20	21.3
Give them a written copy of the websites name and address, show them the website homepage on a computer at the veterinary clinic	2	2.1
Show them the website homepage on a computer at the veterinary clinic	8	8.5
Tell them the name or address of a particular website	30	31.9
Tell them the name or address of a particular website, give them a written copy of the websites name and address	16	17.0
Tell them the name or address of a particular website, give them a written copy of the websites name and address, show them the website homepage on a computer at the veterinary clinic	15	16.0
Tell them the name or address of a particular website, show them the website homepage on a computer at the veterinary clinic	3	3.2

Total	94	100

#### Impact on Vet–Client Relationship

More veterinarians reported feeling that clients’ use of the Internet for pet health information has had a negative impact on the vet–client relationship (54%) compared to those who felt it has had a positive impact (37%). Furthermore, 40% of veterinarians stated that the pet health information on the Internet has had a negative impact on the health of companion animals, with 37% reporting a positive impact and the remaining 23% stating it has had no impact on companion animal health. Regarding time spent with a client, approximately half of respondents stated that the Internet has had no impact on how much time they spent with clients (51%) with the remaining (49%) stating they have needed to spend more time with clients. No veterinarians felt that the Internet had reduced the amount of time they need to spend with clients.

## Discussion

This study found that most UK veterinarians feel their clients access the Internet to find pet health information, yet often do not understand what they read online. This is a similar finding to Kogan et al. (4) who found that for US veterinarians, 63.5% perceived that 0–40% of clients understand online information, compared to 73% in this study. Similarly, Kogan et al. (4) found 62.9% of veterinarians perceived that between 41 and 80% of clients trusted what they read online compared to 72% in this study. Importantly, 40% of veterinarians stated that the Internet has a negative impact on companion animal health. This compares to 23.1% of veterinarians in the US that stated the Internet had a negative impact on pet health (4). This could be due to the wide range of online information sources which are available and with a varying quality of information, especially when this information could involve complex pet health issues. Veterinarians highlighted the concern for pet owners as only 7% of veterinarians thought that between 60 and 100% of clients understand what they read online. Previous studies have found that online pet health information can lack accuracy and completeness (3, 6, 7). This may be further complicated through client search strategies, as veterinarians may use and/or suggest specific reliable pet health websites in mind whereas clients may simply search through search engines and pick sites with minimal additional information. To understand this in more detail further research would need to investigate the reason for this response from veterinarians and could be incorporated into a larger study.

This may also highlight the importance of veterinary information prescriptions as veterinarians can provide suggested websites to clients and help them sources the most reliable and up to date information. However, this study found that only a small number of veterinarians provide online information to clients. Further research needs to be conducted to assess the impact (e.g., patient outcome measures, length of veterinary visits, etc.) of information prescriptions in the UK, similar to Kogan et al. (8), in the US, who found information prescriptions to have a positive impact on clients and their decision about their pet and encouraged discussion with their veterinarian.

It is important that these similarities are interpreted with caution due to the small sample size and convenience sampling method used in the current study. In addition, there may be a range of differences between the UK and US which may be apparent, such as demographics and differences in number of veterinarians. For example in the US, it has been reported that there are over 106,000 veterinarians compared to under 20,000 in the UK (10, 11). While frequency of client type and the purpose of visit (e.g., health problem, frequency of visit, client insurance, information source) may differ between the US and UK. Further research is also need to highlight the similarities and difference between these countries.

Research on clients’ actual use of the Internet and their associated perceptions is a next logical step. The authors are conducting a follow up study investigating UK pet owners’ self-reported behaviors (e.g., frequency of online use and online search strategies to source pet health information) and perceptions (including the likelihood of use of website provided by veterinarians). Overall, this small-scale study found mixed opinions regarding veterinarians’ perceptions of their clients’ use of the Internet and the potential impact it has on the client–veterinarian relationship. Many comments by respondents spoke to concerns they have about the potential negative impact of the Internet, an area which has recently been highlighted. For example, one respondent stated “*Online misinformation is a major cause of stress and frustration to myself & colleagues*” while another veterinarian stated that “*Most clients who do bring their Internet results/searches up are at this stage looking for professional confirmation at this stage. I believe this may become more of a challenging scenario with time*.” In contrast positive aspects were also noted with a respondents stating “*I find that Dr Google is sometimes a very useful tool in supporting my advice and helps the client understand sometimes complex problems at their leisure*.”

It is important to highlight the limitations of this study including small sample size (equivalent to the approximately 0.5% of the practising veterinarians in the UK) and method of distribution. Having said this, Internet surveys have been found to target a wide range of people who may not be reached with traditional survey methods, can be distributed and completed in a timely manner and is cheap to administer (12). Furthermore, additional avenues were targeted, including two veterinary blogs and a veterinary magazine (9) to recruit a broader range of veterinary professionals that solely those who use more popular social media platforms. The use of online sources to obtain participants might have impacted the type of respondents and their views and familiarity with the Internet. A further more detailed large-scale study would be useful which uses a mixed method survey combining both paper-based and online surveys to target a broader range of veterinarians.

## Author Contributions

All authors listed have made a substantial, direct and intellectual contribution to the work, and approved it for publication.

## Conflict of Interest Statement

The authors declare that the research was conducted in the absence of any commercial or financial relationships that could be construed as a potential conflict of interest.
